# Sustained release ivermectin-loaded solid lipid dispersion for subcutaneous delivery: *in vitro* and *in vivo* evaluation

**DOI:** 10.1080/10717544.2017.1284945

**Published:** 2017-03-10

**Authors:** Mengmeng Lu, Dan Xiong, Weiwei Sun, Tong Yu, Zixia Hu, Jiafeng Ding, Yunpeng Cai, Shizhuang Yang, Baoliang Pan

**Affiliations:** The Department of Veterinary Parasitology, College of Veterinary Medicine, China Agriculture University, Hai Dian District, Beijing, China

**Keywords:** Ivermectin, sustained release, solid lipid dispersion, subcutaneous delivery, physicochemical properties, bioavailability

## Abstract

This work aimed to develop a sustained release solid dispersion of ivermectin (IVM-SD) in a lipid matrix (hydrogenated castor oil, HCO) for subcutaneous delivery. Solvent-melting technology was employed to prepare IVM-SDs using HCO. The physicochemical properties of the IVM-SDs were evaluated by scanning electron microscopy (SEM), X-ray powder diffraction (XRPD), and Fourier transform infrared spectroscopy (FTIR). The release of IVM from IVM-SDs was evaluated with HPLC *in vitro.* Pharmacokinetics of IVM was studied in rabbits following a single subcutaneous administration of IVM-SD formulations. The efficacy of IVM-SD against the ear mange mite was evaluated in rabbits. IVM was completely dispersed in HCO in an amorphous state at a drug:carrier ratio lower than 1:3. No chemical interactions between drug and carrier were found besides hydrogen bonding for the amorphous IVM-SDs. The amorphous IVM-SDs formulations exhibited a sustained release of IVM versus physical mixtures (PMs) of IVM and HCO. The drug release decreased as the drug:carrier ratios decreased, and the release kinetics of IVM were controlled via diffusion. Cytotoxicity of IVM-SD to MDCK cells was lower than native IVM. The IVM plasma concentration of SD1:3 remained above 1 ng/mL for 49 d. Higher AUC, MRT, and *T*_max_ values were obtained at a SD1:3 relative to the IVM group. The IVM-SD improved almost 1.1-fold bioavailability of drug compared with IVM in rabbits. IVM-SD could provide longer persistence against rabbit’s ear mites than a commercial IVM injection. This study shows that these solid lipid dispersions are a promising approach for the development of subcutaneous IVM formulations.

## Introduction

Ivermectin (IVM) is a semi-synthetic substance derived from avermectin, which is naturally produced by *Streptomyces avermitilis*. It is very effective in the control of endo- and ecto-parasites such as gastrointestinal nematodes, lice, and mites in livestock (Camargo et al., 2010). Since its commercial introduction for the treatment of parasitic diseases in animals in the 1980s, over 5 billion doses of IVM have been sold worldwide making it the most commonly used antiparasitic drug in animals (Omura, 2008). IVM is still used to treat billions of livestock and pets around the world and increases the production of meat and leather products. IVM is also used for the treatment of parasitic diseases in humans. Since it was first approved to treat onchocerciasis (river blindness) in humans in 1988, IVM has been used worldwide to treat a variety of internal nematode infections including onchocerciasis, filariasis (elephantiasis), and strongyloidiasis as well as ectoparasitic infections by lice and mites.

However, the oral bioavailability of IVM is very low – especially in ruminants. This is because of its poor water solubility, binding to organic materials in the gastro-intestinal tract, and transport by the P-glycoprotein present in the intestinal epithelium. As a result, IVM is generally administered subcutaneously to maximize its bioavailability. A few novel drug delivery technologies and systems have been used to develop the injectable IVM formulation (Rothen-Weinhold and Dahn, 2000; Dong et al., 2014). Here, we prepared a sustained release injectable IVM formulation through solid dispersion (SD).

Sustained release formulations have several advantages over conventional dosage forms. They provide a uniform and prolonged therapeutic effect and reduce the frequency of dosing. In livestock, controlled release formulations of endectocides are effective to limit pasture contamination, reduce parasite transmission, and prevent re-infection. Sustained release formulations also save time and labor. Moreover, they are easily adopted by farmers (Rehbein et al., 2013). Several approaches have been used to develop sustained release dosage forms, including solid lipid nanoparticles, lipid micro-particles, and SD (Sahoo et al., 2009; Camargo et al., 2010; Xie et al., 2010; Rosiaux et al., 2014). The SD technique was originally developed to improve the dissolution properties and bioavailability of poorly water-soluble drugs by dispersing them into solid matrix carriers (Kohri et al., 1999; Chokshi et al., 2007; Kennedy et al., 2008; Oh et al., 2011; Yan et al., 2012). Recently, the dissolution retardation of drug through SD techniques using hydrophobic matrices (lipid-based or non-lipid-based) for the development of controlled release formulations has become an active area of research (Tetsuya Ozeki et al., 2005; Varshosaz et al., 2006; Tran et al., 2011; Shah et al., 2012). The SD technique has become an attractive approach for controlling drug release. The structure of SD is monolithic where drug molecules homogeneously disperse, and it has a great advantage of avoiding the risk of a burst release of drug concerning the reservoir-type preparations (Giri et al., 2012).

Interestingly, lots of attentions have been paid to oral formulation (e.g. tablets), while efforts on injectable formulations have lagged (Tanaka et al., 2005; Giri et al., 2012). Compared with oral formulations, injectable formulations offer no first-pass elimination and no negative effects from food on absorption of drug. Most importantly, injectable formulations generally show a better potential in keeping drug release for longer period than oral formulations. For example, in screening an IVM slow-release formulation for malaria vector control, a slow-release oral tablet could only increase the time with plasma concentrations above LC_50_ of IVM for mosquitoes in hours, while an injectable, depot formulation could do it for weeks; a silicone-based subcutaneous formulation was successful for months (Lo et al., 1985; Chaccour et al., 2015).

To demonstrate that solid lipid dispersions are a useful tool to develop injectable and sustained release formulation, a solid lipid dispersion of IVM (IVM-SD) was prepared with a lipid matrix – hydrogenated castor oil (HCO). The HCO was chosen because it is a readily biodegradable wax and has been used as a sustained-release skeleton material in pharmaceutical formulations (Xie et al., 2011). The HCO is also affordable and can reduce the cost of formulations for veterinary use.

First, IVM-SDs were prepared by solvent-melting technology. Then the physicochemical properties of IVM-SD were evaluated by scanning electron microscopy (SEM), X-ray powder diffraction (XRPD), and Fourier transform infrared spectroscopy (FTIR). The dissolution profiles were studied to understand the mechanism of IVM-SDs release behavior. The cytotoxicity of IVM-SD was evaluated on MDCK cells. The pharmacokinetics of IVM-SD and its efficacy against mites were studied in laboratory rabbits.

## Materials and methods

### Chemical agents

IVM was purchased from Hebei Veyong Animal Pharmaceutical Co., Ltd. (Shijiazhuang, China). HCO was supplied by Tongliao Tonghua Castor Chemical Co., Ltd. (Tongliao, China). HPLC grade methanol was purchased from Merck KGaA (Darmstadt, Germany). Ethyl acetate and other regents were purchased from China National Pharmaceutical Group Corporation (Sinopharm, Beijing, China). The reference standard of IVM (96.0%) was purchased from Dr. Ehrenstorfer GmbH (Augsburg, Germany).

### Preparation of IVM formulations

The SD of IVM (IVM-SD) was prepared by the solvent-melting method. Briefly, 30 g of IVM was dissolved in 30 mL ethyl acetate. The appropriate amount of HCO was then melted at 100 °C before the IVM solution was added. The mixture was stirred at 100 °C for 15 min to remove ethyl acetate and then was immediately cooled at −20 °C for 12 h to form a SD. Subsequently, the IVM-SD was dried under vacuum at 35 °C for 48 h to remove the remaining solvent. Then the IVM-SD was pulverized and sieved to obtain IVM-SD 150–180 μm powder.

Five IVM-SDs at different drug:carrier weight ratios were prepared (1:1, 1:2, 1:3, 1:5, and 1:7, named as SD1:1–SD1:7). Five physical mixtures of IVM and HCO (IVM-PMs) at the same drug:carrier weight ratios (named as PM1:1–PM 1:7) were prepared by mixing and pulverizing the appropriate amounts of IVM and HCO in a mortar. The physical mixtures were sieved and collected as described for IVM-SD above.

### Determination of drug loading and encapsulation rate

The concentration of IVM in the IVM-SDs was determined by HPLC. Briefly, the IVM-SD powder was dissolved in chloroform. Afterwards it was diluted with methanol and filtered through a 0.22 μm filter. It was then analyzed by HPLC (Shimadzu Co., Ltd, Tokyo, Japan) with a UV detector at 245 nm (SPD-M20A). The mobile phase of methanol/water (95/5, by volume) was eluted at a flow rate of 1.0 mL/min through a reverse phase C18 column (250 mm*4.6 mm, i.d., 5 μm). The column temperature was set at 37 °C. The assay was performed in triplicate. The drug loading (DL) and encapsulation rate (EA) were defined as follows:

$$\mathrm{DL}=\frac{\mathrm{weight}\;\mathrm{of}\;\mathrm{IVM}\;\mathrm{in}\;\mathrm{SD}}{\mathrm{weight}\;\mathrm{of}\;\mathrm{SD}}\times 100\%$$

$$\mathrm{EA}=\frac{\mathrm{weight}\;\mathrm{of}\;\mathrm{IVM}\;\mathrm{in}\;\mathrm{SD}}{\mathrm{weight}\;\mathrm{of}\;\mathrm{IVM}\;\mathrm{added}\;\mathrm{in}\;\mathrm{preparation}\;\mathrm{of}\;\mathrm{SD}}\times 100\%$$

### Particle morphology

The shape and surface characteristics of IVM, IVM-SDs, IVM-PMs, and HCO particles were observed by SEM (S4800, Hitachi, Tokyo, Japan).

### XRPD

The powder X-ray diffraction patterns of the samples were obtained by an X-ray diffractometer (D8, Bruker AXS GmbH, Karlsruhe, Germany) using Cu-Kα radiation. Samples were scanned at 2*θ* angle over a range of 3–40°at 4°/min with a step size of 0.02°.

### FTIR

The drug–carrier interactions were measured with a PerkinElmer Spectrum 100 FT-IR Spectrometer (PerkinElmer Inc, Waltham, MA). Samples of each IVM formulation were thoroughly mixed with potassium bromide at 1:10 and pressed at 10 tons for 3 min. The materials were scanned by FTIR at 400–4000 cm^−1^ with a resolution of 2 cm^−1^.

### *In vitro* drug release kinetics

The *in vitro* release behavior of the IVM-SDs was determined using the nylon bag method analogous to the dialysis bag method. The IVM-SD preparations (containing 200 mg of IVM) were placed into a 50 μm mesh nylon bag and immersed in 50 mL phosphate buffer pH 7.4 containing 0.2% (w/v) sodium dodecyl sulfate (SDS-PBS). The 0.2% SDS (a surfactant) was added to maintain the sink condition.

The release assay was performed at 37 °C with continuous agitation at 180 rpm. At regular intervals, 2 mL of medium was withdrawn and filtered through a 0.45 μm Millipore filter. The withdrawn sample was replaced with 2 mL of fresh SDS-PBS to maintain a constant volume. The filtrate was diluted 20-fold with methanol. IVM concentration was measured by HPLC as described above. The *in vitro* release behavior of IVM-SDs was compared with IVM-PMs and native IVM.

To evaluate the mechanism of drug release, the experimental data were fitted to the following kinetic models. The first-order model equation (1) describes release of a substance from a system where release rate is concentration dependent. The Higuchi model equation (2) describes the release of drugs from insoluble matrix as a square root of time dependent process based on Fickian diffusion. The zero-order model equation (3) describes drug release kinetics where the drug release rate is independent of its concentration. The Hixson–Crowell model equation (4) describes the release rate depending on the change in surface area and particle diameter.

First-order model

(1) $$\ln\left(1-\frac{M_{t}}{M_{\infty }}\right)=-\mathrm{kt}$$

Higuchi model

(2) $$\frac{M_{t}}{M_{\infty }}={\mathrm{kt}}^{1/2}$$

Zero-order model

(3) $$\frac{M_{t}}{M_{\infty }}=\mathrm{kt}$$

Hixson–Crowell model

(4) $$\sqrt[{3}]{M_{\infty \;}}-\sqrt[{3}]{M_{\infty }-M_{t}}=\mathrm{kt}$$

### *In vitro* cytotoxicity studies

Cytotoxicity of IVM-SDs was evaluated in MDCK cells using a MTT assay (C0009, Beyotime, Jiangsu, China). The cells were seeded into a 96-well microplate at a density of 1 × 10^4^ cells per well with 0.1 mL DMEM supplemented with 10% fetal bovine serum (FBS) and antibiotics, and were cultured in a 5% CO_2_ incubator at 37 °C. After 24 h, the medium was replaced by 100 μL DMEM complete medium containing SD1:3 (final drug concentration: 5, 10, 25, 50, 100, or 200 μg/mL). Native IVM at the same concentration and carrier (HCO) at the same amount was included as controls. After 24 h of co-incubation, the cell viability was assessed by MTT assay. Typically, 5 mg/mL of MTT were added to 10 μL to each well and incubated for 4 h. Then the formazan liquid was added. The absorbance was read at 570 nm by a microplate reader. Cells along with cell culture media were kept as negative control; cell culture media only was used as the positive control. The cell viability (%) was calculated as follows:

$$\mathrm{Cell}\;\mathrm{Viability}\left(\%\right)=\frac{{\mathrm{OD}}_{\mathrm{test}}-{\mathrm{OD}}_{\mathrm{positive}\;\mathrm{con}.}}{{\mathrm{OD}}_{\mathrm{negative}\;\mathrm{con}.}-{\mathrm{OD}}_{\mathrm{positive}\;\mathrm{con}.}}\times 100\%$$

### Pharmacokinetic study in rabbits

New Zealand rabbits (2.3–3.1 kg) were obtained from Experimental Animal Center of Changyang Xishan Farm, Beijing, China. The animals were housed at 20 ± 2 °C with free access to diet and water. They were acclimatized for one week before treatment.

Eight male New Zealand Rabbits were randomly allocated to two groups (four rabbits per group). IVM was administrated subcutaneously (2 mg/kg of body weight) in the form of either IVM or IVM-SD (SD1:3) suspended in sterilized double-distilled water with appropriate amount glycerol. The animals were managed under a protocol approved by the Laboratory Animal Institute of China Agricultural University.

Blood samples (1 mL) were collected from the marginal ear vein into heparin-coated tubes at 2, 4, 8, and 12 h and 1, 1.5, 2, 3, 5, 7, 10, 14, 21, 28, 35, 42, and 49 d after dosing. The blood samples were centrifuged immediately at 4000 rpm for 20 min, and the plasma was collected and stored at −20 °C until analysis. The concentration of IVM was determined by HPLC following a previously described method with minor modifications (Xu et al., 2007). Briefly, IVM in plasma was extracted with methanol, and puriﬁed with ODS C18 SPE cartridge (Supelco, Bellfonte, PA). The elution was collected and evaporated to dryness with a stream of nitrogen. Derivatization of IVM in dried residue was conducted with *N*-methylimidazole, triﬂuoroacetic anhydride (Sigma Chemical, St Louis, MO) and acetic acid. Then mobile phase was added, and 20 μL of the solution was injected into the chromatograph. The concentrations of IVM were determined by HPLC system (Waters e2695, Milford, MA) with fluorescence detection (Waters 2475, Milford, MA). Separation was carried out on sunfire C18 column (150 mm*4.6 mm, i.d., 5 μm) at 35 °C. The mobile phase of methanol/acetonitrile/water (65.5/30/4.5, v/v/v) was pumped at a ﬂow rate of 1.0 ml/min. The detector was fixed at an excitation wavelength of 365 nm and an emission wavelength of 475 nm. The pharmacokinetic parameters of IVM were calculated using a non-compartmental model with software Phenix WinNonlin5.2 (Pharsight Corporation, Mountain View, CA). All results were expressed as mean ± SD (*n* = 4).

### Efficacy of IVM-SD against the ear mange mites in rabbits

To assess the efficacy of IVM-SD, 21 female rabbits naturally infected with *Psoroptes cuniculi* were used. The *P. cuniculi* ear mange mite was targeted in this study because it is one of predominant parasites in both laboratory and commercial rabbits, and re-infection often occurs during its control with multiple treatments often required to completely eliminate it. The rabbits were housed under a protocol approved by Laboratory Animal Institute of China Agricultural University within Beijing Animal Use and Care Association. The animals were individually kept in wire cages equipped with automatic watering devices and feeders containing commercial rabbit food pellets. Food and water were available ad libitum. These animals were assigned ranks on the basis of lesion scores and then were allocated to groups one to three according to lesion scores so that each group contained rabbits with a similar range of lesion severity. Each group was kept in a separate room. Group one (eight rabbits) and group two (eight rabbits) were subcutaneously administrated with SD1:3 (2 mg/kg) and commercial IVM injection (Ivomec® at 0.2 mg/kg). Group three (five rabbits) was administrated only with vehicle as a control. The animals were examined on days 0 (prior to treatment), 7, 14, 28, and 42. At each examination, the type of lesion was recorded, and ear scabs were collected. The extent of lesions was scored as 0–6 following the literature (Nong et al., 2013). The ear scabs were observed microscopically to evaluate the presence of mites.

### Statistical analysis

The results were analyzed using a one-way ANOVA followed by the HSD test using the SPSS20.0 software (IBM SPSS Statistics, Chicago, IL). A *p* value of 0.05 or less was considered significant.

## Results and discussion

### Drug loading and encapsulation rate

The drug-loading values of IVM-SDs were 46.3–12.3%, and the encapsulation rate of IVM from the SD preparations was over 90% (see in the Supplementary data, Table S1), indicating that most of the drug was encapsulated in the particles. These results showed that the solvent-melting method used in the present study was an effective method to prepare IVM-SD (Singh and Pathak, 2013).

### Particle morphology

The native IVM under SEM is crystalline with regular and smooth surfaces and edges. The HCO has rough surfaces and irregular edges. The IVM-PMs were a simple mixture of IVM and HCO, and the IVM crystals and HCO were clearly visible (Figure S1). In contrast, the crystalline form of IVM was not seen in IVM-SDs; all particles exhibited rough surfaces and irregular edges suggesting that IVM was dispersed in the carrier (HCO) in the SD preparations. The SEM analyses illustrated that the IVM-SDs were homogeneous. Good homogeneity of IVM-SDs is crucial to ensure homogeneity of developing formulation with IVM-SDs.

### XRPD

**Figure 1.**
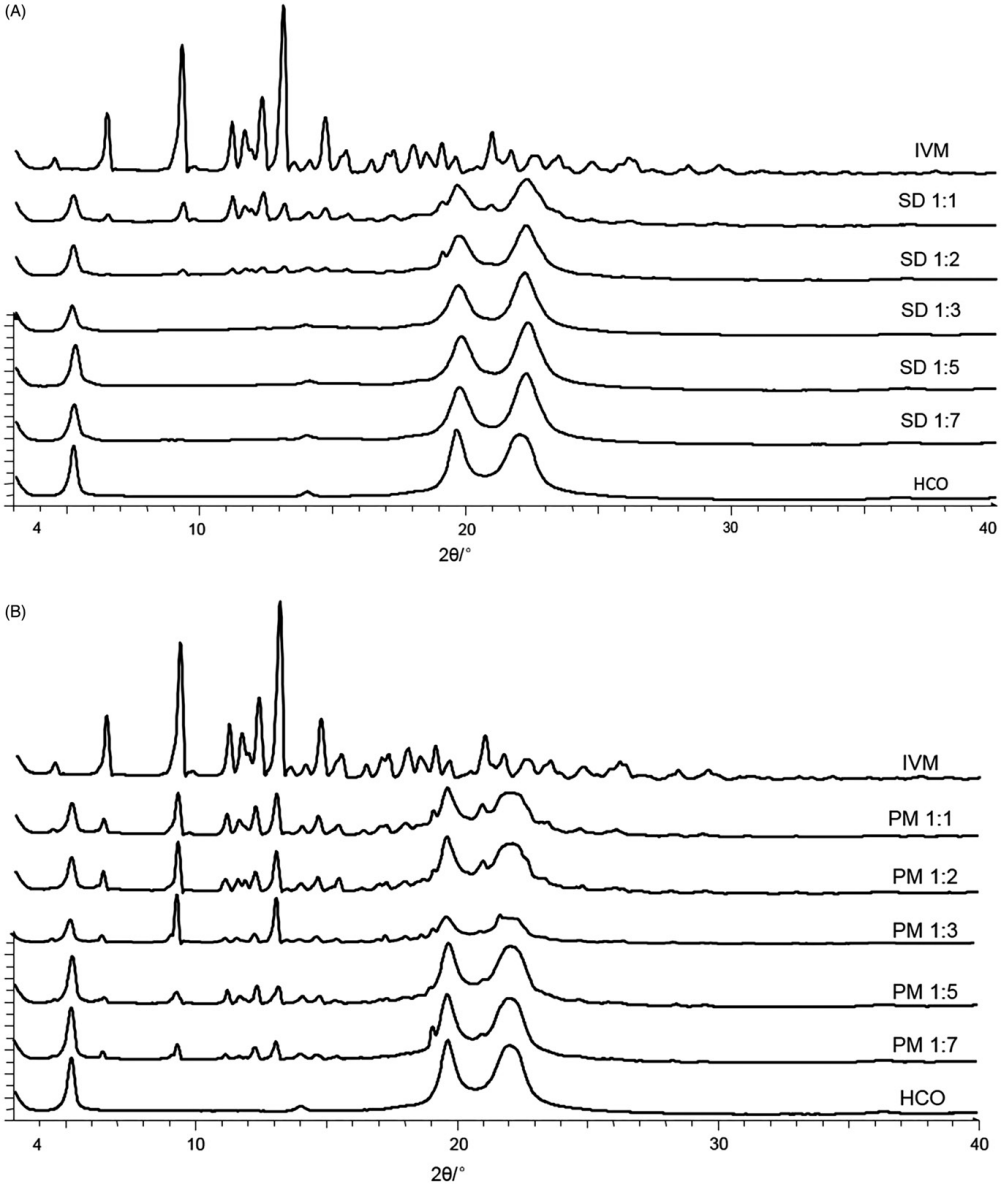
X-ray powder diffraction patterns of IVM-SDs, IVM-PMs, IVM, and HCO. IVM-SD, ivermectin-loaded solid dispersion; IVM-PM, physical mixtures of IVM, and HCO; IVM. native ivermectin; HCO, hydrogenated castor oil, the carrier of solid dispersion.

The native IVM had a typical crystalline pattern with several characteristic sharp peaks at diffraction angles of 20.97°, 19.08°, 14.69°, 13.10°, 12.31°, 11.65°, 11.18°, 9.29°, 6.47°, and 4.48° (Figure 1). The HCO showed characteristic peaks at 22.01°, 19.65°, and 5.20°. The XRPD patterns of IVM and HCO were similar to those described by other authors (Yang and Hrymak, 2011; De Meirleir et al., 2013; Rolim et al., 2015). As expected, the physical mixtures (IVM-PMs) showed diffraction peaks consistent with the presence of crystalline IVM, and the peak intensity of crystalline IVM decreased as the HCO ratio increased (Figure 1B).

In the diffractogram of SD1:2, the intensity of crystalline peaks of IVM was low indicating poor crystallinity of the IVM in SD1:2 (Figure 1A). Characteristic peaks of crystalline IVM were clearly seen in the diffractogram of the SD1:1 indicating that crystalline IVM exists in SD1:1. This demonstrated that the drug was overloaded in the SDs at high drug-to-carrier ratios of 1:1 and 1:2.

As observed by other authors, when an excessive amount of the drug is loaded into a SD system, a small portion of drug molecules is dispersed monomolecularly, some molecules exist in an amorphous state, and a higher amount of drug than its solubility in the carrier dispersion is in a crystalline state (Okonogi and Puttipipatkhachorn, 2006). The SDs of SD1:3, SD1:5, and SD1:7 showed no typical peaks of IVM indicating that IVM was in an amorphous state in these SD preparations. The XRPD data correlated with the SEM results.

### FTIR

**Figure 2.**
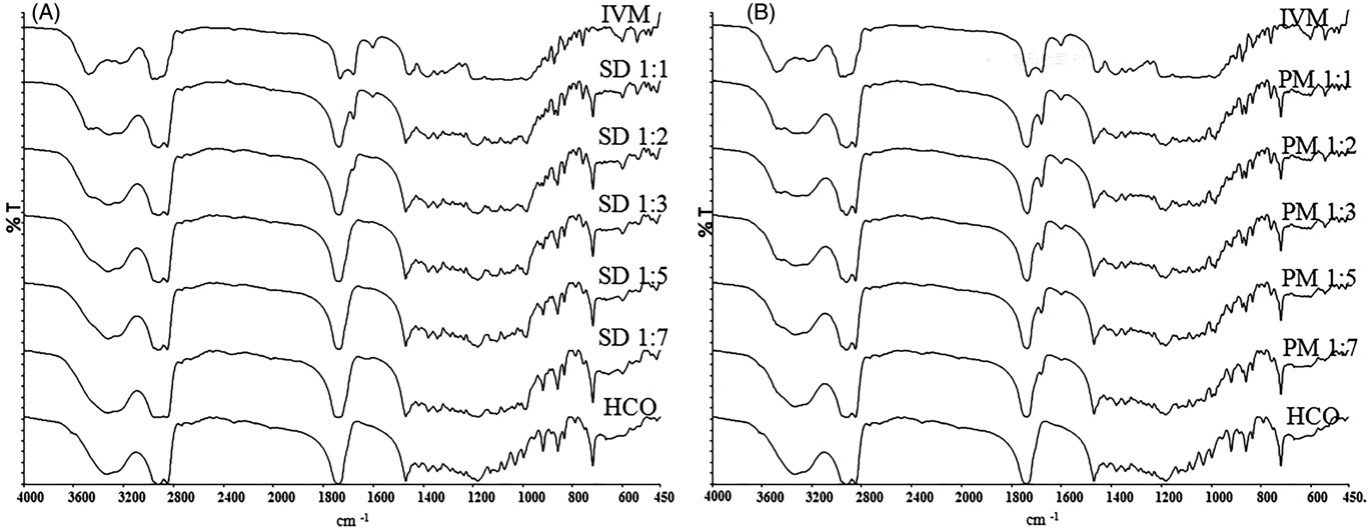
Fourier-transformed infrared spectroscopy spectra of IVM-SDs, IVM-PMs, IVM, and HCO. IVM-SD, ivermectin-loaded solid dispersion; IVM-PM, physical mixtures of IVM, and HCO; IVM, native ivermectin; HCO, hydrogenated castor oil, the carrier of solid dispersion.

FTIR studies were carried out to investigate the interaction between IVM and HCO (Figure 2). Native IVM showed sharp, characteristic peaks (cm^−1^) at 3482.58 due to axial deformation of free O–H; at 2965.59, characteristic of methyl groups; at 2935.16, indicating axial deformation of C–H; at 1732.12, characteristic of saturated aliphatic ketone C=O stretching; at 1678.9, relating to unsaturated lactones with a double bond adjacent to the –O– group, owing to the C=C group; at 1383.87–1313.85, indicating moderate absorption of ketones; and at 1183.43–1024.55 showing the aliphatic ethers due to the asymmetric axial deformation of C–O–C.

Spectral analysis of HCO showed characteristic peaks at 3338.04 cm^−1^ relating to free OH-stretching vibration, at 2922.72 and 2849.88 cm^−1^ characteristic of methyl groups, and at 1738.02 cm^−1^ relating to characteristics of saturated aliphatic ketones. These values agreed well with previously reported results (Shah et al., 2012; Singh and Pathak, 2013; Rolim et al., 2015).

The major IVM peaks were retained in the spectrum of IVM-SDs and IVM-PMs. However, for SD1:3, SD1:5, and SD1:7, there was a slight shift in the C=O group peak of HCO (from 1738.02 to 1736.88 cm^−1^) (Figure 2A). The C=O group peak of IVM (1732.12–1736.88 cm^−1^) resulted in a broader peak of the C=O group. This result suggested hydrogen bonding between the carbonyl group of HCO and the O–H groups of IVM. Apart from these findings, the peak at 1678.9 cm^−1^ (C=C stretch) vanished in the spectrum of amorphous IVM-SDs (SD1:3, SD1:5, and SD1:7). However, the 1678.9 cm ^−1^ peak was present in all the IVM-PMs (Figure 2B). This suggested that the presence of carbonyl-containing HCO disrupts the unsaturated lactones dimer of IVM resulting in hydrogen-bonding association between the carbonyl group of HCO and O–H groups of IVM. Amorphous drug exists in a higher free energy state and their tendency to recrystallize, while the interactions between the carrier and drug will prevent the drug from recrystallizing (Thiry et al., 2016). These interactions could enhance the physical stability of amorphous IVM-SDs. The absence of new, unidentified peaks indicated no drug–carrier chemical interactions, which was confirmed by XRPD. No chemical interactions were seen between IVM and HCO in IVM-SD. This is essential for the safety of IVM formulation based on IVM-SD because the chemical interaction between drug and carrier can possibly increase the toxicity of drug.

### *In vitro* drug release

**Figure 3.**
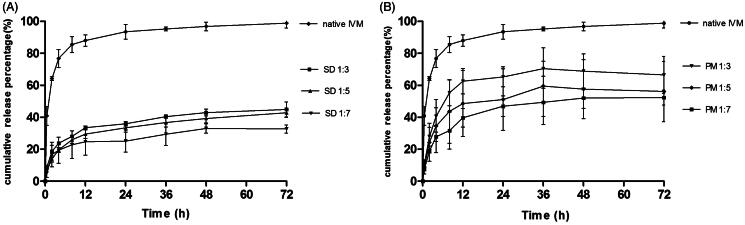
*In vitro* dissolution profile of IVM-SDs, IVM-PMs, and IVM. IVM-SD, ivermectin-loaded solid dispersion; IVM-PM, physical mixtures of IVM, and HCO; IVM, native ivermectin; HCO, hydrogenated castor oil.

The *in vitro* release profiles of native IVM, IVM-SDs, and IVM-PMs were studied in 0.2% SDS-PBS. The cumulative drug release over 72 h was 98.9% for native IVM from 44.93 to 32.74% for IVM-SDs (Figure 3A) and from 66.54 to 52.36% for IVM-PMs (Figure 3B). The initial release of IVM-SDs was less than 9% within 0.5 h without burst release and the release profile of IVM-SDs kept a sustained increasing. Sustained release of drug and no burst release are two cornerstones for the development of controlled release formulations. The IVM-SDs exhibited a slower release versus IVM-PMs at the same drug:carrier weight ratio. The percent release and the release rate of IVM from IVM-SDs decreased with increasing drug:carrier ratio indicating that HCO controlled the drug release from IVM-SDs. Interestingly, the IVM-PMs also exhibited a lower percent drug release and release rate versus native IVM. This might be due to the highly hydrophobic HCO, which made it difficult for the medium to penetrate into the IVM particles (Shah et al., 2012). The *in vitro* release assay demonstrated sustained-release of IVM from the SD formulations.

The release data were fitted to different kinetic models to describe the kinetics of drug release from the matrix and to understand the release mechanism of IVM from IVM-SDs. The Higuchi model gave the highest regression coefficient (R^2^) for all amorphous SD formulations (see Table S2) indicating that Higuchi model would be most suitable for modeling the release of IVM from amorphous SD formulations. These results suggested that IVM was released from the SD by diffusion. For a drug with a diffusion-controlled release mechanism, its release is mainly governed by the carrier.

Sun et al. demonstrated that medium-insoluble carriers (such as ethylcellulose and hydroxypropyl methylcellulose acetate succinate at pH 1.2) facilitated a diffusion-controlled release mechanism of drug from SDs. In this study, HCO – a medium-insoluble carrier – also mediated a diffusion-controlled release mechanism of IVM from SDs resulting in a sustained release of IVM from SDs (Figure 3). The release rate of IVM decreased with increasing proportion of HCO in the IVM-SDs. The SEM and XRPD results showed that IVM was completely dispersed in HCO in IVM-SDs. The hydrophobic HCO coating on the IVM-hindered medium penetration and drug diffusion and hence retarded drug release. These results indicated that the drug release was mainly governed by HCO, and the drug release rate could be adjusted by changing the drug:carrier ratios. The carriers only act as the vehicles in SDs. High concentrations would increase the possible toxicity of formulation and impose the burden for body. Although HCO is a biodegradable and safe carrier, the minimum use in IVM-SDs is desirable (Xie et al., 2011; Li et al., 2012). Therefore, the SD1:3 was chosen for the cytotoxicity, pharmacokinetics, and efficacy studies in rabbits.

### Cytotoxicity

The cytotoxicity study showed results in MDCK cell lines with SD1:3 (IVM concentration from 5 to 200 μg/mL) and native IVM and HCO (Figure S2). The carrier (HCO) showed no cytotoxicity at each concentration on MDCK. SD1:3 and native IVM showed no cytotoxicity at 5 and 10 μg/mL, respectively. With increasing doses, the SD1:3 and native IVM displayed an increased cytotoxicity; however, the survival percent of cells was greater than 50% in all SD1:3 groups, whereas the native IVM showed much higher cytotoxicity than SD1:3 particularly when the concentration of IVM was more than 25 μg/mL. These results indicated that the SD1:3 (the IC_50_ value was 184.67 μg/mL) had far lower toxicity toward MDCK cells than native IVM (the IC_50_ was 26.65 μg/mL). Molinari et al. (2009) demonstrated that native IVM showed significant cytotoxicity to Chinese hamster ovary (CHOK1) cells when the concentration was higher than 50.0 μg/mL. The difference of cytotoxicity of IVM between two studies may be due to different cell lines and different experimental conditions.

### Pharmacokinetics of IVM-SD in rabbits

**Figure 4.**
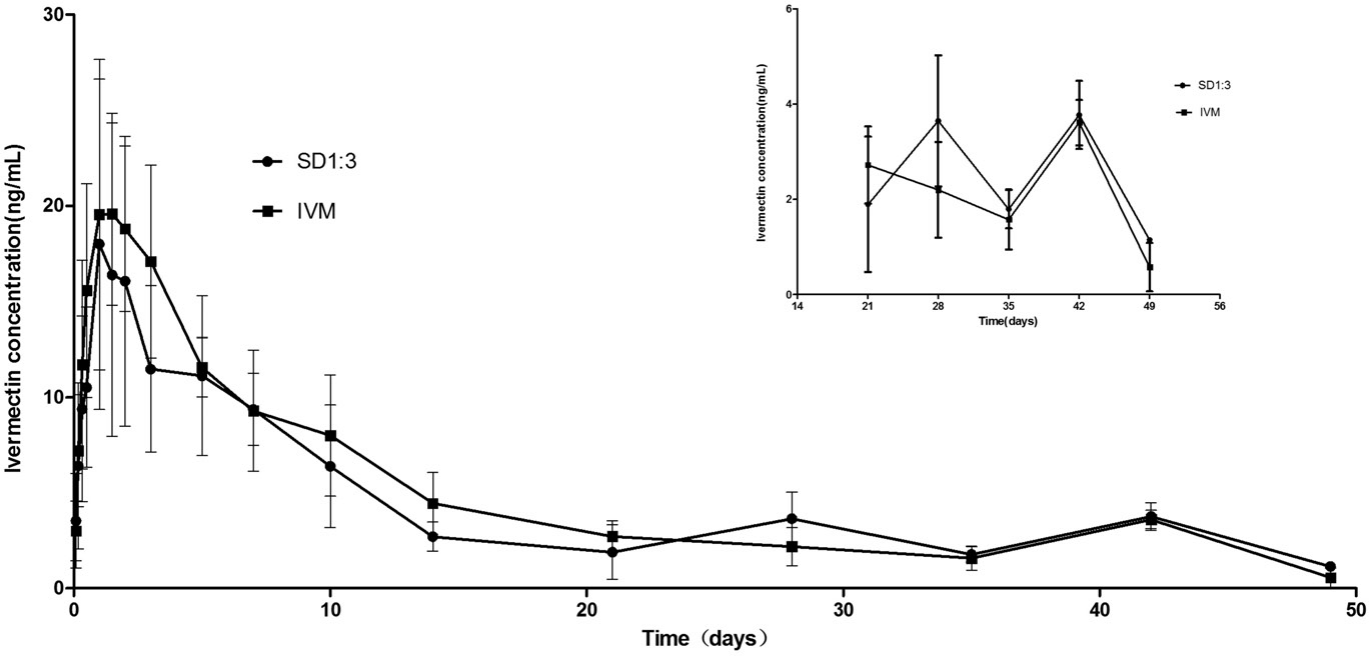
Plasma time–concentration profiles of IVM after subcutaneous administration of SD1:3 and IVM in rabbits. The inset figure was the plasma time–concentration profiles after 21 d. The results were expressed as mean ± SD (*n* = 4). SD1:3, ivermectin-loaded solid dispersion with drug:carrier weight ratios of 1:3; IVM, native ivermectin.

The pharmacokinetics of the SD formulation were studied in rabbits by subcutaneous injection. The plasma concentration of IVM kept above 1 ng/mL for the entire sampling period of 49 d in the SD1:3 group (Figure 4). The time (49 d) was remarkably longer than those of Ivomec® formulation and the liposomal formulation of IVM (about 8 d) (Bassissi et al., 2006). Furthermore, the SD formulation exhibited significantly longer mean residence time (MRT) (30.31 d) than native IVM (18.45 d) (Table 1). The MRT for SD1:3 was longer than those for Ivomec® formulation (2.75 d) and the liposomal formulation of IVM (2.24 d) (Bassissi et al., 2006). The MRT is an integrated measure of all processes governing the fate of a drug in the body (absorption, distribution, and elimination) and is the most pertinent parameter for comparing drug persistence in the body. These results indicated that the sustained release of IVM from SD1:3 could be maintained under physiological conditions. Furthermore, AUC and *T*_max_ of IVM for SD1:3 (277.6 ng d/mL and 2.75 d) were slightly higher than IVM (255.61 ng d/mL and 1.5 d). The SD1:3 enhanced the subcutaneous bioavailability of IVM as reflected by AUC_INF_ by 1.1-folds. The enhancement of bioavailability of sustained release IVM-loaded solid lipid dispersion will prolong therapeutic effect and reduce the frequency of dosing.

**Table 1.** Main pharmacokinetic parameters (mean ± SD, *n* = 4) of IVM after subcutaneous administration of SD1:3 and IVM in rabbits.

Parameters	IVM	SD1:3
*C*_max_(ng/mL)	22.25 ± 5.97	18.18 ± 8.53
*T*_max_ (day)	1.50 ± 0.58	2.75 ± 2.87
AUC_INF_ (ng day/mL)	255.61 ± 68.63	277.6 ± 84.91
MRT (day)	18.45 ± 4.97	30.31 ± 5.61^a^

The letter “a” denotes significant differences between the SD1:3 and the IVM group (*p* < 0.05).

Interestingly, there was no significant difference between SD1:3 and native IVM in their pharmacokinetic profiles (Figure 4). However, attractive properties could be observed in a close observation of the profile of IVM-SD (Figure 4, inset figure). From about 24 d (observed from the Figure 4) to the end of study, a higher plasma concentration of IVM was seen in the SD1:3 group than in the native IVM group. Higher plasma drug concentrations generally imply higher efficacy. At the end of the study, the IVM level in plasma in the SD1:3 group was still above 1 ng/mL (the minimal drug level required for optimal activity for most parasites), but not in the native IVM group. Based on the pharmacokinetics of SD1:3, we predicted that IVM-SD would provide higher efficacy and longer persistence against parasites than native IVM. Furthermore, in our later study, native IVM showed worse potential in making a stable suspension. It was difficult to mill native IVM to the required sizes, and the IVM particle in the suspension had a strong tendency to aggregate with poor re-dispersibility. Therefore, native IVM was not included in our further studies including the efficacy study.

According to the results, the amorphous formulation of SD1:3 with high drug loading and sustained persistence *in vivo* is a good candidate for the development of long-acting injectable IVM formulations.

### Efficacy assessment in rabbits

The lesion scores and the number of mite positive rabbits were recorded during the therapeutic test (Table 2). All rabbits treated with SD1:3 (2 mg/kg body weight) had no mites by the 14th day and remained negative until the end of the study (42 d). All rabbits in this group clinically recovered by the 28th day after treatment. Although Ivomec® showed rapid and high efficacy against ear mites, e.g. no mites were observed in treated rabbits on the 7th day after treatment, it failed to prevent reinfection, e.g. one rabbit was re-infected on the 14th day after treatment and five were re-infected on the 42nd day. For untreated rabbits, the infection remained, and the lesions became serious with deteriorating rabbit health. Considering the animal welfare, the rabbits in the untreated group were treated with SD1:3 on the 14th day. This also resulted in a clinical and parasitological cure on the 42nd day.

**Table 2.** Clinical scores of infection of rabbits and number of positive rabbits.

		Days				
Groups	Efficacy criteria	0	7	14	28	42
SD1:3	Number of positive rabbits	8	2	0	0	0
	lesion scores	2.63 ± 1.30^a^	0.63 ± 0.23^a^	0.44 ± 0.18^ab^	0.00 ± 0.00^a^	0.00 ± 0.00^a^
(IVOMEC)	Number of positive rabbits	8	0	1	3	5
	lesion scores	2.13 ± 1.25^a^	0.44 ± 0.18^a^	0.86 ± 0.94^b^	1.58 ± 1.24^b^	2.25 ± 0.99^b^
Vehicle Control	Number of positive rabbits	5	5	5	0	0
	lesion scores	2.00 ± 0.71^a^	3.20 ± 0.45^b^	3.40 ± 0.55^c^	0.30 ± 0.27^a^	0.00 ± 0.00^a^

The different letters within a column denote significant differences between the different groups (*p* < 0.05).

This efficacy study demonstrated that a single dose of SD1:3 at 2 mg/kg following subcutaneous administration could eliminate *P. cuniculi* infection in rabbits. It is well known that IVM has no ovicidal effects on the eggs of mite, and IVM in the body of animals cannot affect mites off of the host. Normally, the eggs of *P. cuniculi* hatch in 4 d (Flynn, 1973). Mites off host remain viable and infectious for approximately 15 d (O'Brien et al., 1994). Both newly hatched larvae and mites off host can become sources of reinfection leading to repeat treatment needed in the control of *P. cuniculi* with conventional formulations of IVM, doramectin, and selamectin (Bowman et al., 1992; Voyvoda et al., 2005; Mellgren and Bergvall, 2008). The absence of mites in rabbits from the 14th to the 42nd day after subcutaneous administration of SD1:3 indicated that sufficient drug concentrations were available to kill any larvae hatching from eggs as well as to prevent re-infection of mites off host. Therefore, no repeat treatment is necessary in the control of *P. cuniculi* with IVM-SD. However, the results showed that multiple administrations of Ivomec® were needed to prevent reinfection of the ear mites in rabbits. The re-infected rabbits were treated at the end of the study.

## Conclusion

In summary, sustained release SDs of IVM were prepared with a lipid carrier (HCO) using a solvent-melting method. The IVM was completely dispersed in the carrier in an amorphous state in the IVM-SDs when the drug:carrier ratio was lower to 1:3. There were no chemical interactions other than hydrogen bonding between IVM and HCO. Sustained release of IVM from IVM-SDs was observed *in vitro*. Drug release from the SD was controlled by diffusion and governed by the ratio of IVM:HCO. The SD1:3 showed lower cytotoxicity to MDCK cells than native IVM. The SD1:3 could keep the plasma drug level above the minimal effective concentration for more than 49 d following a subcutaneous administration. A single dose of IVM-SD (SD1:3) at 2 mg/kg following subcutaneous administration could completely eliminate *P. cuniculi* infection in rabbits. This study shows the SD techniques using lipid matrix (HCO) as a carrier are a promising approach for the development of sustained release formulations of IVM for subcutaneous delivery. Studies on the long-term stability of IVM-SD and the development of the injectable suspension are underway.

## Supplementary Material

Table_S2._Regression_coefficient__R2__of_release_profiles.docx

Table_S1.The_Drug_loading_and_encapsulation_rate_of_IVM-SDs.docx

Figure_S2._Cytotoxicity_of__SD13__HCO_and_IVM__on_the_MDCK_cell_lines.jpg

Figure_S2._Cytotoxicity_of__SD13__HCO_and_IVM__on_the_MDCK_cell_lines.docx

Figure_S1._Scanning_electron_microscopy_photomicrographs.jpg

Figure_S1._Scanning_electron_microscopy_photomicrographs.docx

## Notes

**Supplementary material available online**
