# Supplementary material for: Sustained release ivermectin-loaded solid lipid dispersion for subcutaneous delivery: in vitro and in vivo evaluation
Source: Drug Deliv. 2017 Mar 10;24(1):622–31. doi: 10.1080/10717544.2017.1284945 (PMC8240974; doi:10.1080/10717544.2017.1284945)
Supplement: Table_S2._Regression_coefficient__R2__of_release_profiles.docx [file IDRD_A_1284945_SM9905.docx]

| Formulation | Regression coefficient value (R^2^) | | | |
| --- | --- | --- | --- | --- |
|  | Higuchi | First order | Zero order | Hixson-Crowell |
| SD1:3 | 0.9655 | 0.9072 | 0.8108 | 0.8977 |
| SD1:5 | 0.9882 | 0.9349 | 0.8666 | 0.9284 |
| SD1:7 | 0.8593 | 0.7451 | 0.7234 | 0.7355 |
| PM1:3 | 0.9836 | 0.9619 | 0.8828 | 0.945 |
| PM1:5 | 0.9609 | 0.9088 | 0.8384 | 0.8926 |
| PM1:7 | 0.9626 | 0.9136 | 0.8503 | 0.9027 |
| IVM | 0.8904 | 0.8999 | 0.6196 | 0.8546 |

Table S2. Regression coefficient (R^2^) of release profiles based on four release models.
