# Supplementary material for: Sustained release ivermectin-loaded solid lipid dispersion for subcutaneous delivery: in vitro and in vivo evaluation
Source: Drug Deliv. 2017 Mar 10;24(1):622–31. doi: 10.1080/10717544.2017.1284945 (PMC8240974; doi:10.1080/10717544.2017.1284945)
Supplement: Table_S1.The_Drug_loading_and_encapsulation_rate_of_IVM-SDs.docx [file IDRD_A_1284945_SM9902.docx]

Table S1.The Drug loading and encapsulation rate of IVM-SDs (mean± SD, n=3))

| Samples | Drug loading  (actual) | Encapsulation rate |
| --- | --- | --- |
| SD1:1 | 46.3%±1.08% | 90.59%±2.11% |
| SD1:2 | 31.3%±0.39% | 97.54%±1.21% |
| SD1:3 | 24.21%±0.86% | 98.76%±3.49% |
| SD1:5 | 15.96%±0.18% | 96.14%±1.08% |
| SD1:7 | 12.3%±0.12% | 98.16%±0.93% |
