# Supplementary figures and images for: Sustained release ivermectin-loaded solid lipid dispersion for subcutaneous delivery: in vitro and in vivo evaluation
Source: Drug Deliv. 2017 Mar 10;24(1):622–31. doi: 10.1080/10717544.2017.1284945 (PMC8240974; doi:10.1080/10717544.2017.1284945)

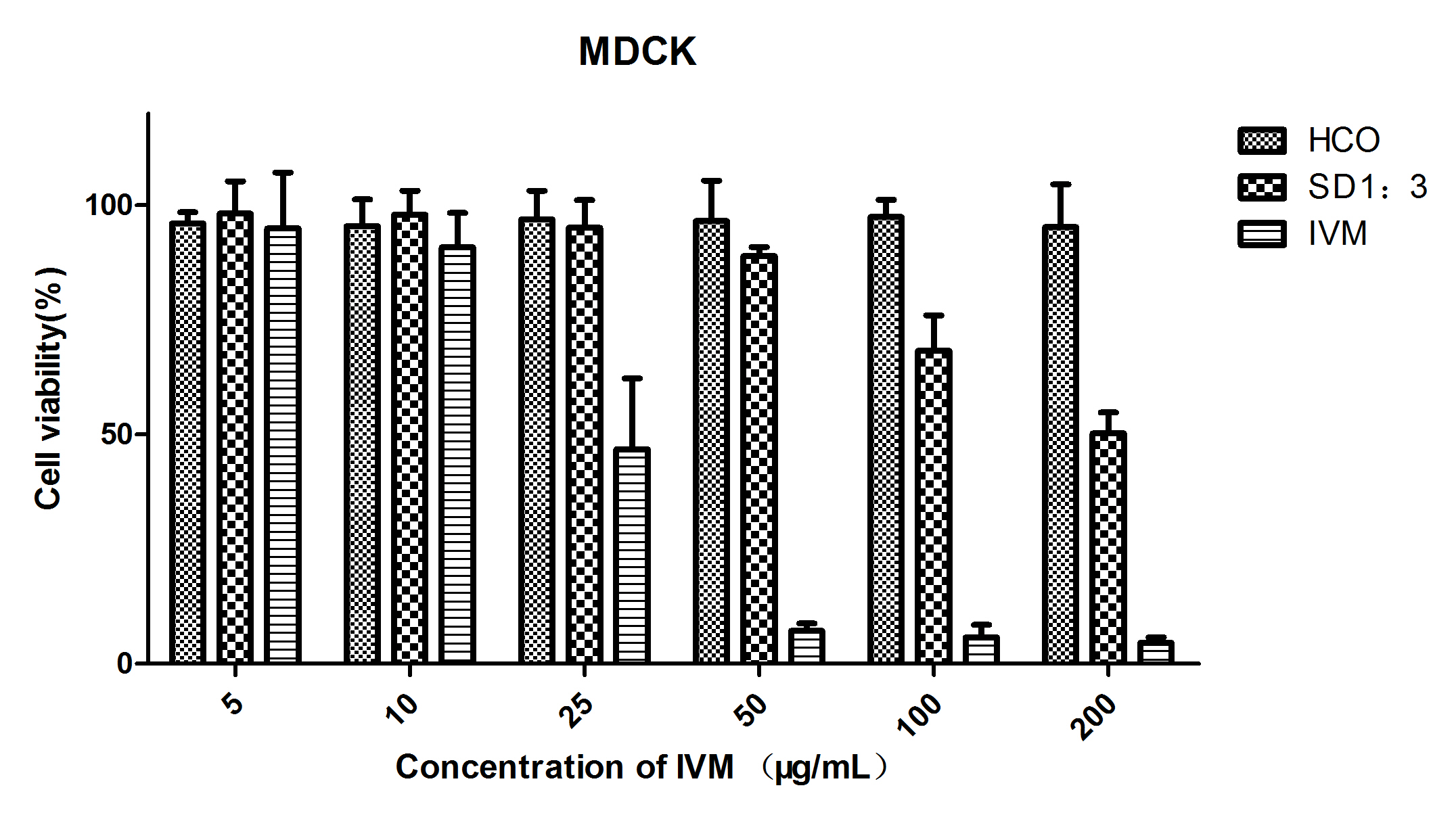

Supplement: Figure_S2._Cytotoxicity_of__SD13__HCO_and_IVM__on_the_MDCK_cell_lines.jpg [file IDRD_A_1284945_SM9898.jpg]

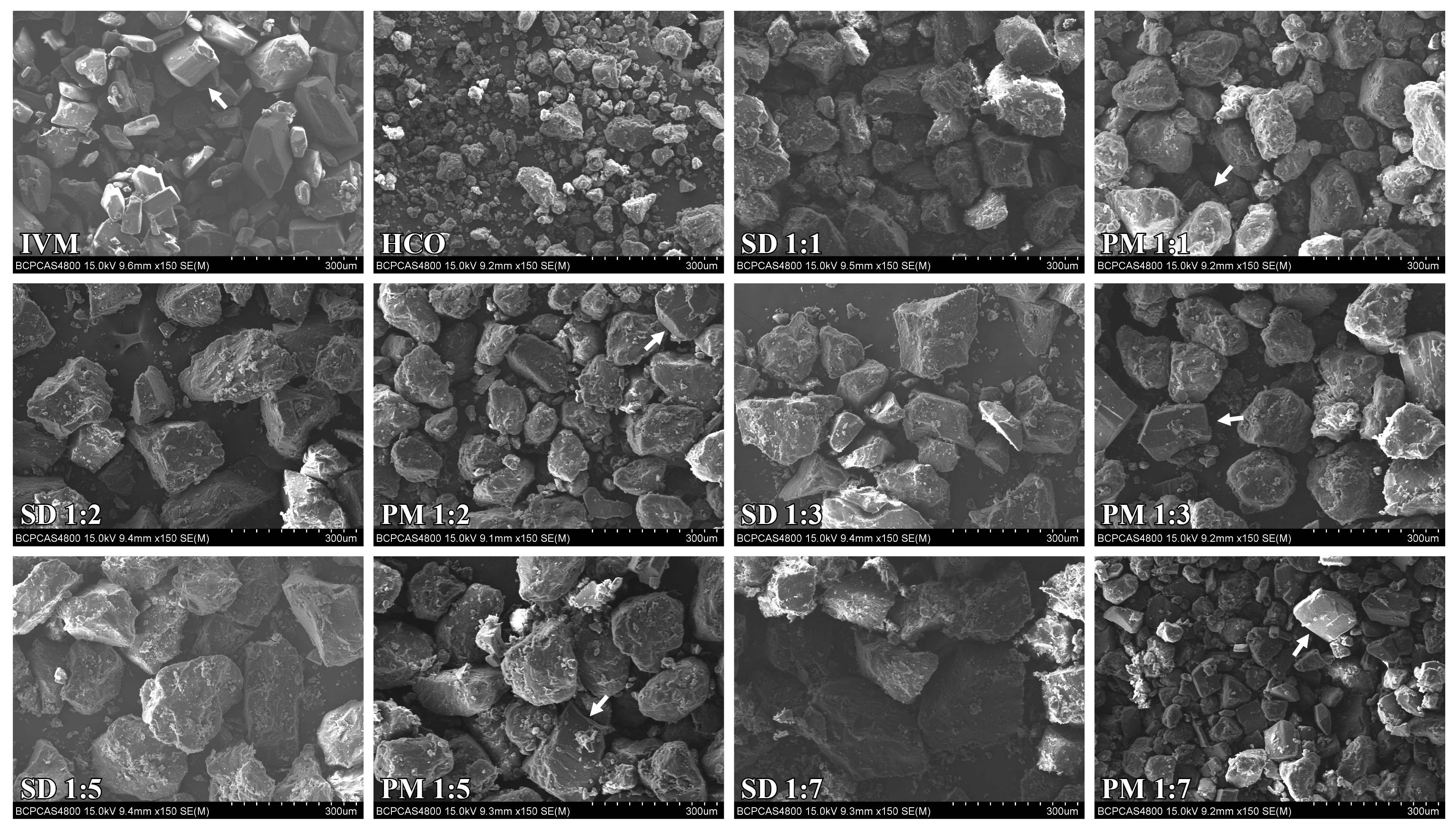

Supplement: Figure_S1._Scanning_electron_microscopy_photomicrographs.jpg [file IDRD_A_1284945_SM9886.jpg]
