# Supplementary material for: Sustained release ivermectin-loaded solid lipid dispersion for subcutaneous delivery: in vitro and in vivo evaluation
Source: Drug Deliv. 2017 Mar 10;24(1):622–31. doi: 10.1080/10717544.2017.1284945 (PMC8240974; doi:10.1080/10717544.2017.1284945)
Supplement: Figure_S2._Cytotoxicity_of__SD13__HCO_and_IVM__on_the_MDCK_cell_lines.docx [file IDRD_A_1284945_SM9894.docx]

Figure S2. Cytotoxicity of SD1:3, HCO and IVM on the MDCK cells. The results were expressed as mean ± SD (n = 4). SD1:3, ivermectin-loaded solid dispersion with drug:carrier weight ratios of 1:3; HCO: hydrogenated castor oil, the carrier of solid dispersion; IVM: native ivermectin.
