# Supplementary material for: Sustained release ivermectin-loaded solid lipid dispersion for subcutaneous delivery: in vitro and in vivo evaluation
Source: Drug Deliv. 2017 Mar 10;24(1):622–31. doi: 10.1080/10717544.2017.1284945 (PMC8240974; doi:10.1080/10717544.2017.1284945)
Supplement: Figure_S1._Scanning_electron_microscopy_photomicrographs.docx [file IDRD_A_1284945_SM9865.docx]

Figure S1. Scanning electron microscopy photomicrographs of IVM, HCO, IVM-SDs, and IVM-PMs. IVM-SD: ivermectin-loaded solid dispersion; IVM-PM: physical mixtures of IVM and HCO; IVM: native ivermectin; HCO: hydrogenated castor oil, the carrier of solid dispersion. Note: IVM crystal is indicated by a white arrow.
